# From bean collection to seed bank: transformations in heirloom vegetable conservation, 1970–1985

**DOI:** 10.1017/bjt.2019.2

**Published:** 2019-06-14

**Authors:** Helen Anne Curry

**Affiliations:** Department of History and Philosophy of Science, University of Cambridge, Free School Lane, Cambridge, CB2 3RH, UK

## Abstract

In 1975, the Missouri homesteaders Kent and Diane Ott Whealy launched True Seed Exchange (later Seed Savers Exchange), a network of ‘serious gardeners’ interested in growing and conserving heirloom and other hard-to-find plant varieties, especially vegetables. In its earliest years, the organization pursued its conservation mission through member-led exchange and cultivation, seeing members’ gardens and seed collections as the best means of ensuring that heirloom varieties remained both extant and available to growers. Beginning in 1981, however, Kent Whealy began to develop a central seed repository. As I discuss in this paper, the development of this central collection was motivated in part by concerns about the precariousness of very large individual collections, the maintenance of which was too demanding to entrust to most growers. Although state-run institutions were better positioned to take on large collections, they were nonetheless unsuitable stewards because they placed limits on access. For seed savers, loss of access to varieties via their accession into a state collection could be as much an ending for treasured collections as total physical loss, as it did not necessarily enable continued cultivation. As I show here, these imagined endings inspired the adoption of a new set of conservation practices that replicated those seen in the formal genetic conservation sector, including seed banking, cold storage and safety duplication.

## Introduction

In 1975, the Missouri homesteader Kent Whealy dispatched a first circular of the True Seed Exchange to its twenty-nine founding members. The bulk of this six-page document, which had been ‘copied on an unguarded Xerox machine at Boeing Aircraft in Wichita, Kansas’, consisted of extracts of letters sent by members to Whealy detailing the garden varieties for which they had seeds to share and the varieties they wished to obtain from other growers.^1^ Whealy had gathered these individuals via a letter published in a handful of back-to-the-land publications, in which he invited correspondence from people maintaining treasured varieties in their gardens. ‘If you’ve been gardening for a few years and are keeping seeds that you *know* – from your own personal experience – run true, send me your name and address and what kinds of seeds you’ll have’, he wrote. This would be the basis of a list that Whealy would circulate by post to anyone who sent money to cover printing and mailing costs. Subscribers could, in turn, use the list to correspond with and obtain seeds from other ‘serious gardeners in similar climates’.^2^ Not all seeds were welcome. The ‘true seed’ referred to in the exchange’s name were specifically those that were not the F1 hybrids often sold by seed companies, as these would not grow ‘true’ from saved seed.^3^ As Whealy elaborated in subsequent mailings, he wanted members with ‘heirlooms’ or ‘old, reliable, superior vegetable varieties’ to share, those stewarding ‘vegetable seeds which have been passed down over generations’.^4^ Gathering these was, in his view and that of his wife and cofounder of the True Seed Exchange Diane Ott Whealy, a matter of urgency: ‘As our older gardeners pass on and their seeds are not replanted, we lose genetic strains every bit as valuable and irreplaceable as any other endangered species on our planet … We must find and spread these heirloom vegetable varieties as quickly as possible.’^5^

Whealy professed disappointment in only having twenty-nine members the first year, but membership quickly grew, to 141 members in 1976 and more than three hundred by 1978.^6^ Through the publication of an annual newsletter containing the list of members and their seeds (soon large enough to be a bound book) that was edited by Whealy and initially funded by a modest subscription fee, these scattered gardeners and farmers were united into a network that facilitated conservation through exchange.^7^ Each winter, members assessed their stock of seeds saved from previous harvests and wrote to Whealy with a ‘has’ list detailing the varieties they had on hand and could offer to others. Many also included a list of ‘wants’ as well, describing varieties they hoped to obtain through exchange. By 1981, Whealy boasted that ‘approximately 600 different members have offered an estimated 3,000 heirloom or unusual vegetable varieties to over 9,000 interested gardeners’ via the newsletter. According to his ‘conservative’ estimation, ‘150,000 plantings have been made of vegetable varieties that aren’t in any seed catalog and in many cases were on the edge of extinction’.^8^ A name change announced in 1979 explicitly celebrated this act of salvation. As Whealy explained to members, the organization’s new name, Seed Savers Exchange, ‘more accurately reflects who we are and what we are doing. We are all SEED SAVERS and we are literally saving seed of old vegetable varieties from extinction’.^9^

The Whealys’ interest in saving heirloom vegetable varieties from extinction blossomed amidst concerns from many quarters about the loss of crop plant diversity, especially the disappearance of locally adapted crop varieties in the wake of agricultural industrialization.^10^ Numerous institutions and systems for the preservation of plant genetic diversity took shape or were reshaped amidst these concerns. In the early 1970s, state-funded efforts focused especially on the creation of secure seed storage facilities, also called seed banks or gene banks. These were institutions that could assure the long-term preservation of diverse varieties of important economic crops along with continued access to these by breeders and other professional researchers.^11^ A number of local and grass-roots initiatives like Seed Savers Exchange also trace their roots to this period.^12^ Like their state-run counterparts, these organizations aimed at long-term preservation, but they typically did so with particular subsets of crop diversity (heirloom, heritage, traditional vegetables and crops) and different sets of users (home gardeners, organic growers, indigenous communities) in mind. In the case of Seed Savers Exchange, these differences initially led the Whealys and their member–collaborators to pursue a conservation strategy distinct from that of state-funded seed and gene banks. Unlike their counterparts at these institutions, the Whealys did not envision centrally managed collections of seeds with hermetically sealed containers and freezer storage to extend the shelf lives of seeds as the way to save endangered vegetables. They opted instead to put in place technologies that would enhance communication among like-minded cultivators. They regarded members’ gardens and personal seed collections as the best means of ensuring that heirloom varieties remained available to current and future gardeners.^13^

This did not remain their only conservation strategy for long. Beginning in 1981, Kent Whealy began to develop a central collection of all the varieties traded through the exchange and a plan for its long-term storage, arguing that the organization’s conservation mission could not be achieved without these. In this paper, I trace the development of this central collection, which soon consumed a considerable portion of the work of Seed Savers Exchange. My account focuses on how ideas about what constituted loss shaped the conservation strategies adopted by the exchange under the direction of its co-founder Kent Whealy. As I show, the loss of varieties imagined or experienced by seed savers – the end of treasured collections – was not always that of absolute physical loss through destruction or decay and death. It could also take the form of loss of access, in which seed savers, by dint of their non-professional status, were typically excluded from using the collections amassed by government institutions. In a sense, state accession had a similar outcome to physical loss: it rendered varieties unavailable to members of the exchange and future heirloom cultivators. The threat of these two imagined endings for collections inspired Whealy to adopt a new set of conservation practices including seed banking, cold storage and safety duplication. These transformed a diffuse and informal grass-roots correspondence network into a centralized conservation operation that replicated important characteristics of its state-funded, professionally staffed national and international counterparts – in the name of providing greater physical security for collections while simultaneously maintaining greater access. Among other outcomes, this technological reconfiguration signalled the alignment of community-led and state seed conservation with respect to the capacity of centralized control and especially cold storage to protect future interests, however different those interests might be.^14^

There is a robust sociological and anthropological literature on ‘seed savers’ – that is, individuals and organizations that participate in or coordinate the local exchange of seeds as a means of ensuring the continuation of so-called traditional, heritage or heirloom varieties.^15^ These studies have highlighted the varied motivations of seed savers, from ‘quiet activism’ that seeks to improve communities through modest acts to more direct political engagement aimed at diminishing the power of agribusiness.^16^ Other contributions have highlighted the unique conservation roles seed savers play in relation to national and international institutions, particularly in attending to varieties that might be overlooked in state institutions and providing a distinct group of cultivators with access to these and other materials.^17^ Here I contribute to this growing literature by highlighting the dynamic historical trajectories of seed savers’ activities: how their motivations and methods changed over time in response to new appreciations of the precarity of individual collections.^18^ Providing such a history, as I do here, complicates a narrative common in the existing literature, in which the approaches of seed savers, focused on exchange and continued cultivation, are set apart from or even in opposition to the typically storage-based strategies of state-led conservation initiatives.^19^ I show instead how some seed savers came to embrace similar methodologies and technologies as were found within state-led programmes, while remaining steadfast in the pursuit of independent goals.

## Ageing gardeners and endangered heirlooms: the origins of Seed Savers Exchange

Essential to understanding what Seed Savers Exchange eventually became is understanding what it initially set out to be. Nearly every account of the origins of Seed Savers Exchange (an organization still in operation today with more than 13,000 members) notes that an important inspiration for the Whealys in launching its forerunner the True Seed Exchange had been seeds of two varieties, a tomato and a morning glory, entrusted to them by Diane Ott Whealy’s grandfather in 1972, shortly before his death. These had come from Bavaria with *his* father in 1870, and he wanted to be sure that someone would keep these family treasures in cultivation. As the story is often told, the Whealys came to recognize that many such varieties might be disappearing as those who had long stewarded them grew older, in all likelihood without access to homesteading grandchildren like themselves to whom the seeds could be passed on.^20^ This inspired the pair to envision a correspondence network that would put seeds of those lines in greater circulation, so that they might be not only continued but also more widely grown and appreciated.^21^ They would encourage the collection, exchange and cultivation of seeds in order to try to avoid the extinction – that is, the total loss – of treasured vegetable varieties.

Accounts less reliably relate a further narrative of the organization’s origins. As Kent Whealy recalled in 1982, and in many of his own retellings of the origins of the exchange network, ‘About the same time [around 1974], I happened to read articles by several scientists, including Dr. Jack Harlan and Dr. Garrison Wilkes, warning about the increasing loss of genetic diversity’.^22^ An article in *Mother Earth News*, a magazine founded in 1970 to cater to the interests and needs of do-it-yourself homesteaders like the Whealys, was particularly influential. The article had been written by the American biologist Paul Ehrlich, and as Whealy recalled, it ‘explained how risky it was for us to be moving toward monoculture plantings and limiting the available varieties of each of our vegetables’. This characterization of a global concern prompted Whealy to reflect on heirloom seed saving as potentially contributing to a much broader set of issues than the continuation of family or community history. ‘I began to wonder just how many other gardeners were – like me – keeping rare or antique seeds … and I could see how important such a hobby could be in combating the situation Ehrlich described’, he remembered.^23^

This narrative suggests that Kent and Diane Ott Whealy fused their interest in heirloom varieties, and concern about the potential loss of family and local treasures, with the growing concerns of agriculturists and plant breeders about the loss of genetic diversity in economic crops and its consequences for global agricultural production. This was not just about ensuring the continued availability of vegetable varieties suitable for small-scale growers to gardeners and homesteaders like themselves. It was also about ensuring that valuable genetic material remained extant and accessible for the long-term future of agricultural production.

Kent Whealy’s description of the mission of Seed Savers Exchange for an early grant proposal offers a particularly clear articulation of this fusion. The proposal had been prepared in the hope that the organization might be able to move away from the shoestring model in which members’ modest subscription fees covered the costs of printing and mailing the annual newsletter. As such, it made a case for the specific and essential contribution of Seeds Savers Exchange to a recognized global issue. After characterizing the concern of ‘the scientific community and laymen around the world’ with ‘the genetic wipe-out of our food crops’, Whealy noted two areas that had yet to receive attention: heirlooms and vegetable varieties being dropped from seed catalogues. With respect to the former, the death of a single seed saver might spell the extinction of unique lines. According to Whealy, ‘Many gardeners keeping heirloom vegetables are very old and their seeds will be lost within years … But when they die, there is often no transfer of seeds or knowledge about their varieties.’ In the case of commercial vegetable varieties, the loss was of perfectly good lines that happened to be deemed not sufficiently profitable. As Whealy described, ‘They are being allowed to die out … with no systematic effort being made by government agencies or lay organizations to keep them alive or store them.’^24^ This void, of course, was where the Whealys and the growing membership of Seed Savers Exchange would step in.

Kent Whealy might well have added to this description that the members of Seed Savers Exchange were also interested in different characteristics for their crops, and hence a different subset of genetic material, than those heading up national and international efforts. The latter tended to emphasize not only crop varieties but also qualities that would be valuable in large-scale agricultural production such as disease resistance, drought tolerance, fertilizer responsivity, suitability for mechanical harvest, and so on. These were, after all, the features most often screened for by the collectors and breeders who contributed to and relied on government repositories. Members of Seed Savers Exchange, by comparison, might share an interest in traits like disease resistance and drought tolerance, but they did not care much about yield; positively detested delayed ripening or fertilizer and pesticide dependence; and sought out specific flavours, colours, cooking qualities, growing habits and even histories.

Some of the varieties that members listed as ‘wants’ in the exchange’s first years give a sense of these diverse needs. In 1976, Lyle Settle sought ‘beans found during Indian pueblo excavations’ as well as a ‘round San Marzano Tomato’. The same year, Howard Jones made a general request for ‘crops that give a quality product with no dependence upon chemical fertilizers’.^25^ In 1978 M.R. Blighton inquired after ‘an Orange Tomato which he grew in about 1942 … [and] believes … was described as “tangerine”. Has tried several including Golden Jubilee, but they don’t come close in quality’. Blighton was accompanied in his quest for better eating qualities by G.A. Hope, who wanted ‘a small amount of Edible Pod English Pea Seed of the wrinkled variety’ because ‘the only ones available from the catalog seed people are smooth seeds which lack the different flavor of the old time wrinkled seed edible pod’.^26^ Memories of varieties once grown by parents and grandparents loomed large, and were often linked to specific traits. Martha Shenan in 1979 wrote in search of ‘a red pepper that her Italian grandmother used to buy in Maine. It was NOT a sweet pepper, but was almost as big as a ripe bell pepper, the same color, but with more lobes, and was FAIRLY hot’.^27^ As these exchange listings illustrate, members of Seed Savers Exchange often sought characteristics (flavours, histories, suitability for home cultivation) that were unlikely to be targeted as priorities for conservation by state and federal institutions in the United States.

This is not to say that those institutions were ineffective. By the early 1970s, the United States had a comparatively robust system for the conservation of crop plant diversity. The US Department of Agriculture (USDA), working in conjunction with state agricultural experiment stations, had inaugurated a system of regional plant introduction stations in the late 1940s. A decade later, in 1958, the US National Seed Storage Laboratory (NSSL) opened in response to calls from professional plant breeders and other agriculturists for a facility that would be solely concerned with the maintenance of genetically diverse plant stocks. By the mid-1970s, these and other US agricultural institutions had been designated as nodes in a National Plant Germplasm System ‘to introduce, maintain, evaluate, catalog, and distribute all types of plant germplasm’.^28^ Not everyone was convinced by the inclusivity of that ‘all’, however. For Whealy and the seed savers with whom he collaborated, the National Plant Germplasm System had shortcomings, including lack of comprehensiveness, which necessitated seed savers’ complementary efforts.

Government institutions were more interested in the varieties desired and maintained by gardeners than Whealy and Seed Savers Exchange members sometimes imagined. The director of the NSSL, Louis Bass, had even described such an interest to Whealy in 1978. ‘We are continually looking for sources of old varieties that are no longer carried in seed catalogs, but are being maintained by individuals for their own use’, he explained.^29^ Equally exaggerated was the characterization of the NSSL as absolutely closed off to individuals without professional status or institutional affiliation. True, NSSL staff typically offered seeds only to what the USDA considered ‘bona fide’ researchers – that is, professional scientists and breeders – and then only when seeds were not available from any other source. They nonetheless appear to have at times sent seeds to precisely the kinds of people who became members of Seed Savers Exchange.^30^ In the early 1970s, NSSL staff not only shared material with John Withee, an enthusiastic bean collector and founder of the heirloom bean exchange Wanigan Associates, but also requested his assistance in growing out and harvesting fresh seed of some ‘old heirloom varieties’ in order to restock the NSSL supplies.^31^

Despite these interconnections, it was nonetheless true that the NSSL and other collections within the USDA conservation system had stringent acquisition policies, as well as limitations on access. It was these shortcomings that Whealy imagined Seed Savers Exchange would address. First, it would fill a gap left by state-run conservation programmes by seeking out and preserving types it believed were overlooked in those systems. Second, it would provide growers shut out of government institutions with access to crop diversity. It thus targeted two kinds of loss, both absolute loss in the form of varietal extinction and the more localized loss of access where seeds survived but were sequestered for a narrow subset of users. Both would be mitigated through the same means: exchange of seeds among like-minded gardeners.

## From seed exchange to seed bank; or, what happens when collectors end?

Within a few years of the launch of Seed Savers Exchange, Kent Whealy began to worry that the exchange was insufficient to ensure successful conservation of heirloom varieties. The death of an elderly member of the exchange in 1978 was an important catalyst to this changing perspective. Burt Berrier, a retired travelling salesmen who had begun gathering seeds on his cross-country sales journeys, was one of what Whealy aptly labelled ‘the Collectors’ – that is, a member who specialized in amassing varieties of a specific crop. Berrier’s preferred crop was beans, which he continued to acquire from friends and correspondents even after his sales days were over. He reported for the anticipated 1978 exchange newsletter that his collection contained 448 varieties, 128 of which he was growing out that season. Berrier had been happy to share this wealth with anyone who wrote to him. His death, therefore, raised concerns about what would happen to his collection. As Whealy recounted, he had written to Berrier ‘and asked if there was some way that our Membership could obtain samples of his collection, so we could multiply and spread them’. Having failed to obtain the beans before Berrier’s death, Whealy wondered whether these lines would be continued.^32^ Working under the assumption that losing saved seeds risked absolute loss of varieties, it seemed possible – likely even – that some of Berrier’s collected varieties would follow him to the grave.

Berrier had harboured worries about this possibility during his lifetime. In search of a future steward of his beans, he appears to have reached out to the NSSL sometime in 1976 to discuss the possibility that these might be incorporated into the national collection. The director of the NSSL, Louis Bass, in turn wrote to the USDA breeders most concerned with bean improvement to see whether they thought this would be useful – and indeed they did. Enquiring further about the Berrier collection, they asked Bass to ‘get some substantial information as to whether they are known cultivars, where they were obtained, and how they were increased, etc’.^33^ In 1977, Berrier wrote to Bass with the information he had on hand about his collection, which was scant in comparison to that which the laboratory wanted and to which it was most likely accustomed:

Have no list of the 200 kinds of beans I have. Most have no names, as they have been sent to me with no information, have found the same bean has different names. Have kept a short record of who and where I got most of them, as to the color of the pod or bean I have no idea, as I’m colorblind. After I’ve grown them I know which is a bush or a pole. Have not grown ½ of them.

Berrier reported having obtained ‘10 kinds from Malawi … 2 beans each’ and similarly ‘a package with some 6 or 7 beans each … from Italy, Spain and Portugal, no information’. He had also ‘got some of the kind that Brigham Young brot with his group from Navo Ill. to S. L. City, Just got them, age unknown’.^34^ Berrier’s passion for collecting beans had evidently derived from an interest that did not necessitate excessive documentation.^35^

Despite Berrier’s poor documentation, the USDA bean breeders remained interested, and in late 1977 Bass made plans to visit Berrier in person to pick up the collection, which by that time had increased to over four hundred varieties. When Berrier died in January 1978, Bass had not yet managed to visit, but Berrier’s wife Maude carried through his intention to donate the collection to the NSSL the following month. In an official letter acknowledging the donation, Bass assured Maude that the future of Burt’s seeds was secure: ‘All materials included in the National Seed Storage collection are maintained indefinitely. Each variety is regrown when necessary and new seed placed in storage in order to maintain a continuous supply of good germinating seed.’^36^

Though it may have been a comfort to Maude, the deposit of her husband’s collection in the NSSL distressed Kent Whealy. As he wrote of these seeds in the 1979 Seed Savers Exchange, ‘Although I appreciated the fact that they were all preserved and protected, I was afraid we had lost access to them.’^37^ In other words, though saved from absolute loss, the types represented in Berrier’s collection appeared to be no more available to exchange members than before. They were, as Whealy acknowledged, still ‘lost’.

Whealy’s fears proved only partially correct. Berrier’s collection – which included assorted seeds from corn to mahogany in addition to the substantial array of beans – was not appropriate for immediate accession into the national system. It arrived packaged mostly in glass baby-food jars, some bearing identifying labels (e.g. ‘Purple Pod’, ‘Cranberry Ohio’, ‘Greasy Indiana’ or ‘Aztex’) and/or a bean glued to the lid as an additional means of identification. The numbers of beans per type, which ranged from two seeds to 550, were far too few to constitute a storage-ready sample (which Bass suggested would be on the order of ten thousand seeds) and in many cases their viability was also in question. In order to be able to share any of them, the laboratory needed to assess and multiply the seed. In the meantime, when Maude Berrier forwarded to Bass seed requests that had arrived after her husband’s death, Bass was obliged to report to those correspondents that the limited quantities and poor condition of some seeds made it difficult for him to pass along items from the collection.^38^

**Figure 1:**
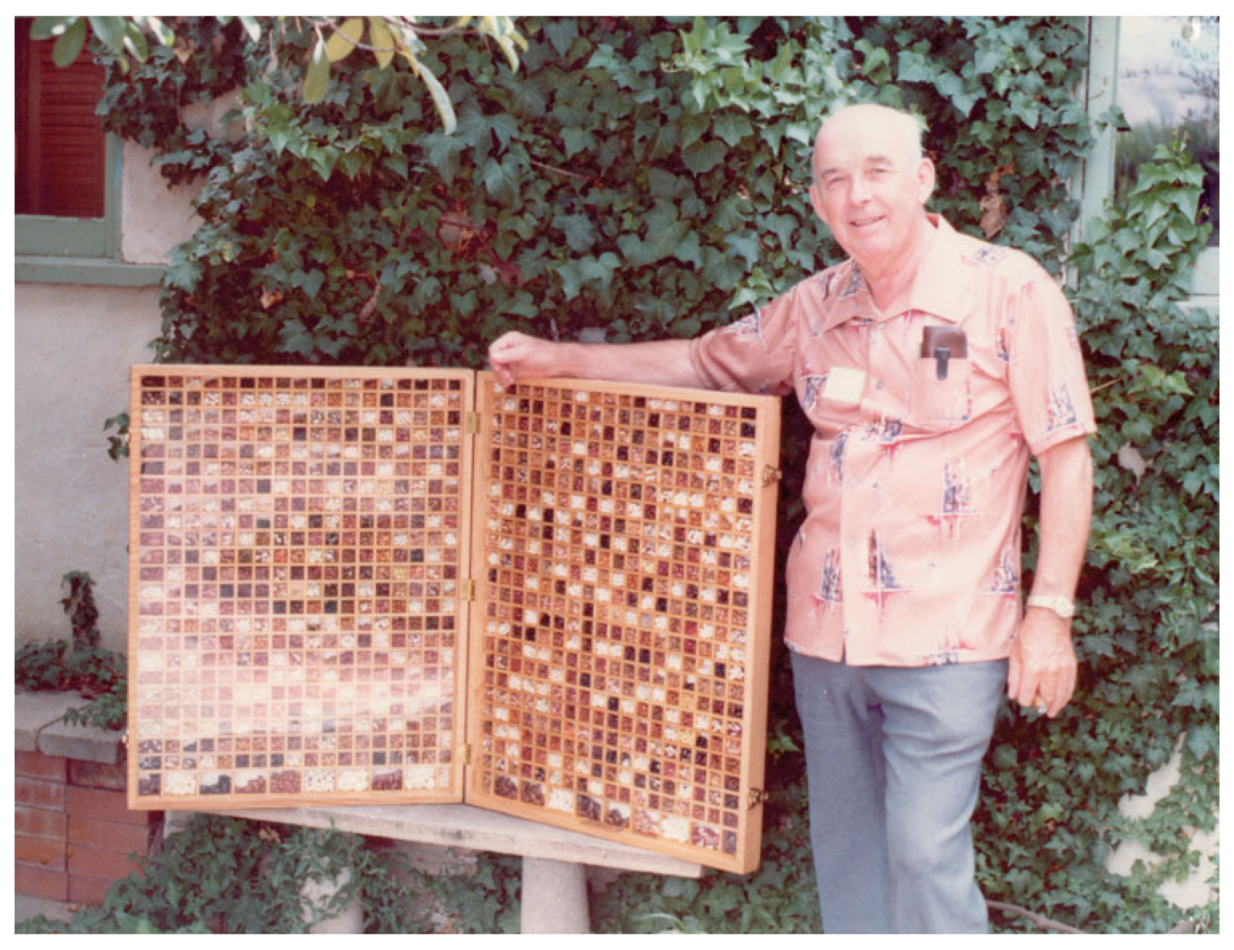
John Withee and his bean display, *c.*1982. Reproduced by permission of Seed Savers Exchange.

As Bass’s response to these inquiries suggests, he was not opposed to the idea of making the beans available to more than just ‘bona fide’ researchers. He was willing, even eager, to collaborate with home cultivators capable of growing out a few varieties and harvesting enough seed that they could augment the NSSL’s supply of these varieties.^39^ Bass successfully orchestrated such an arrangement with John Withee, who had already been engaged to help the NSSL increase some of its bean stocks (see Figure 1). In 1976, Withee had founded Wanigan Associates, a non-profit-making membership organization dedicated to facilitating the collection, cultivation and distribution of heirloom bean varieties. Bass’s vision for increasing and sharing the Berrier collection suggests why Withee must have seemed an ideal collaborator. As Bass wrote to Withee,

If you would be interested in some of these [seeds from Berrier’s collection], I would be glad to send you a few seeds so that they could be added to your collection as well as being included in our program here. By having them in your collection, we could refer individuals to you in the future … Maybe if we keep working together over the years, by exchanging germplasm we can build up a complete collection of heirloom bean varieties for the future.^40^

Withee’s collaboration would make it possible for bean enthusiasts to eventually enjoy access to the collection as much as professional breeders and researchers.

Withee assented to Bass’s plan. However, in making arrangements for exchange, Bass did not offer him access to all of the beans in Berrier’s collection. He instead prepared a list of about eighty varieties, which appear to have been those for which Berrier had provided both a name and a sufficient quantity of seed to share. This represented just a subset of the donated material that Bass seems to have thought viable and worth attempting to regenerate: by comparison, the following year he prepared some 380 samples from the collection for USDA colleagues to attempt to grow out.^41^ Even if the deposit at NSSL had not closed off seed savers’ access to the Berrier collection entirely, there were nonetheless limits on what they could be entrusted with. Where supplies were especially scarce, Bass and his colleagues considered only ‘bone fide’ researchers to be dependable.

Although Whealy later reported to the Seed Savers Exchange that Wanigan Associates had taken on about 50 per cent of the Berrier collection through Withee’s exchange with the NSSL, Withee requested only thirty of the eighty offered by Bass after assessing the gaps in his own collection – and those eighty represented only a subset of the original Berrier collection.^42^ Yet there was still reason to celebrate, as Whealy did, reporting to members of Seed Savers Exchange that the part of Berrier’s collection taken on by Withee would soon be freely available to anyone who joined Wanigan Associates. ‘Burt’s beans’, or some of them, would stay in circulation after all.^43^ The loss of these materials to the community via their accession into the NSSL, tantamount to extinction for home gardeners who could not hope to cultivate these, had not been so complete as he had imagined.

The celebration of this solution – that is, of having Wanigan Associates act as a repository and distribution point for Berrier’s beans – proved short-lived. John Withee, too, was growing older, and the demands of maintaining his huge collection and keeping up with all the correspondence of Wanigan Associates soon seemed like more than he could manage. He needed help or else his collection would be lost, in whole or in part, through the inevitable decay and death of seeds. He turned to the Whealys, asking them to take on what was by then the 1,186 bean varieties of the Wanigan Associates collection.

The Whealys accepted Withee’s proposed transfer of the collection to Seed Savers Exchange, and in so doing they changed the nature of their organization. As its website described in 2017, ‘At the time [1981], Seed Savers Exchange was not a central repository for heirloom varieties. John’s request became the catalyst that led Seed Savers Exchange to become *the largest non-governmental seed bank in the United States*.’^44^ The initial vision of the Whealys had been to create a network of exchange, linking through a central newsletter the many private repositories of genetic diversity that were, typically, members’ ordinary gardens. To this Kent Whealy now added the idea of creating a fail-safe for these private repositories at his family home, which also happened to be the headquarters of the organization. The ‘Heirloom Seed Bank’ as first envisioned by Whealy comprised seed-drying equipment, a tin-canning machine, and a couple of freezers for storage. He proposed in the first instance to gather seeds of varieties that he felt to be most threatened, and here it is telling that he identified the single-crop collections of older members as those in immediate need of attention. In addition to gathering the seeds of these endangered collections on his own, Whealy invited deposits from members of the exchange.^45^ His goal was no less than to reproduce the genetic diversity of the many scattered gardens of his members in a central location, to further safeguard against their loss either through outright extinction or through loss of access to the Seed Savers Exchange network of members. As he explained,

Each year a wealth of germplasm flows through the Seed Savers Exchange, but too much of it never shows up in our Members’ listings the following year. It is hard enough to contact persons keeping heirloom vegetable varieties. For their varieties to be lost after I have contacted them is a tragedy. But that need not ever happen again.^46^

The Whealys had founded Seed Savers Exchange as a way to prevent the extinction of heirloom and other vegetable varieties, but just six years into its operation Kent Whealy had come to see the loose network of exchange among gardeners as, at best, a partial solution. Although membership mostly grew from year to year, individual members came and went, taking their heirlooms with them. Meanwhile, at the opposite end of the spectrum from these drifters, the ultra-dedicated and mostly old-timer collectors amassed such extraordinary collections that it was hard to pass them on to anyone except professionally staffed seed bank facilities. In both cases, seed savers would lose access to varieties – unless, of course, the bank was run by and for those same seed savers.

In subsequent years, Whealy backed off the language of ‘seed bank’. The notion of a central collection remained firm, however, and its accumulation and maintenance were soon the core activities of the Seed Savers Exchange. According to one tally, a steady stream of contributions from a variety of sources led, by 1984, to a collection of some 3,500 varieties, including about two thousand beans (of which about 1,100 had come via Wanigan Associates), five hundred tomatoes, two hundred peppers, 140 corns, and a hundred each of melons, potatoes, lettuces and peas.^47^ When the Whealy family moved from Missouri to Decorah, Iowa, in 1985, the collection moved with them. A central feature of their new property was a regeneration garden set up to maintain the central collection of Seed Savers Exchange. The garden was quickly incorporated as the central feature of a new project, dubbed Seed Savers’ Heritage Farm, and the activity of regenerating the collection in turn became the primary focus of the organization.^48^

## Living and dying in cold storage: convergences in conservation

As he attempted to expand the work of Seed Savers Exchange in the early 1980s, the story of Burt Berrier’s beans proved valuable to Kent Whealy as an example of why his organization was crucial to the cause of conserving crop diversity. A case in point was his less-than-accurate retelling of the history of this collection in a grant proposal of 1981:

Burt Berrier is a good example of what happens when an amateur collector dies. He passed away in January 1978 at the age of 84 and was keeping over 450 varieties of beans that he had collected over a 50 year period … I had been corresponding with Burt for over a year. We were just beginning to make plans to transfer his collection to the membership of the Seed Savers Exchange. Then I heard that he had passed away and that officials from the National Seed Storage Laboratory had picked up his collection. It is the policy of the Laboratory to make available only seed of specific varieties when there is no other known source. So I figured that we had simply lost access to Burt’s collection. But the NSSL’s collections are, for the most part, varieties and relatives of large scale agricultural crops … NSSL is terribly underfunded and understaffed and doesn’t have much money for growing varieties that need multiplying. They store only five pound samples and since almost all of Burt’s samples were smaller than that, they offered the entire collection to John Withee … Approximately 30% of Burt’s 450 varieties had already died due to his decreasing ability to carry the load of his collection during his final years. Out of Burt’s collection, only 180 varieties still survive in the membership of Wanigan Associates.^49^

In assessments like this one, Whealy highlighted the unique contributions of his organization amidst ongoing government conservation efforts like that of the NSSL: securing neglected types and providing access. ‘The type of networks I am developing are satisfying needs that government programs aren’t fulfilling and reaching people not normally reached by them … It is very obvious that a laymen’s exchange of seeds that works as a supplement to government programs is the best plan to pursue’, he declared.^50^ Whealy’s words belied the fact that the ‘laymen’s exchange of seeds’ was itself becoming more like the government programmes from which Whealy distinguished it. He had effectively begun to duplicate the structures and methods of those state initiatives, and would continue in that trajectory in subsequent years.

As efforts at Seed Savers Exchange coalesced around the in-house maintenance of the central collection, Whealy devised ways to share the labour of collection management that in effect repositioned the ‘laymen’s exchange’ as supplemental, rather than central, to the conservation mission. In 1987, he floated the idea of a Network of Curators in which specialist curators would take responsibility (in some cases shared) for all of the varieties of a particular crop that passed through Seed Savers Exchange. They would exchange information centrally, divide up labour in order not to grow the same varieties, and produce annual lists of varieties maintained. Members would be able to consult the lists and contribute seeds of anything they had that was not yet in the care of a curator, thereby adding to the central collection.^51^

The central collection and Network of Curators were seen as necessary to overcome the shortcomings of exchange as a conservation measure – specifically the dubious commitment of many members to long-term engagement. As Whealy described, ‘I think we desperately need to develop this type of maintenance network, because there is just too big a turnover in our membership. There are just too many things that can go wrong.’ If the objective of Seed Savers Exchange was to keep endangered varieties extant and available, and many members could not be counted on to always be growing and especially sharing these varieties year in, year out, the exchange would fail in these underlying objectives. ‘We need to get all of these varieties under wraps and start protecting them and maintaining them in a much more permanent fashion’, Whealy exhorted. ‘If we don’t, five or ten years from now we could discover that all we have really produced is a lot of paper.’^52^ This overriding concern with the potential loss of varieties, through their disappearance from the network, if not necessarily from the world, led Whealy to consider again the necessity of freezer storage – or, as he described it, a ‘frozen back-up collection here at Heritage Farm to protect against the catastrophic destruction of a Curator’s collection’.^53^

Through the 1980s, Seed Savers Exchange pursued a vision of heirloom vegetable conservation in which the underlying network of gardeners, by and large, simply supplied heirloom varieties, whether to other gardeners via the newsletter or to the central collection. This was a role not unlike that of plant explorers in relation to national and international conservation systems.^54^ It was desirable, of course, that they should be seeking out, growing and redistributing these varieties in their own gardens, year after year. Through these efforts, they did the bulk of the work of providing exchange members, old and new, with heirloom varieties – that is, facilitating access. But this distributed, uncoordinated network could not be counted on to keep every single variety in circulation and cultivation, and this, in turn, risked physical loss. Therefore, the central collection at Heritage Farm, maintained by staff of Seed Savers Exchange with the additional help of a number of highly experienced gardeners serving as curators, undertook the core work of ensuring that varieties were conserved for the long term. It is telling of the organization’s altered vision of how conservation was to work that in the event of disaster, the curators and staff of Seed Savers Exchange would be expected to turn not to a member but to a ‘back-up collection’ stored in a freezer.^55^ In short, the conservation mission of Seed Savers Exchange, and the scale of Whealy’s ambitions in this domain, gradually pushed the grass-roots effort in the same direction as the formal genetic conservation sector, in terms of its vision of reliable conservation technologies: they moved from a model of conservation through exchange and cultivation into conservation through centralized curation and safety duplication.

It is possible to gain a more detailed view of how these complementary and convergent conservation systems worked by taking a closer look at the fate of Burt Berrier’s bean collection. How did this treasured collection fare as it travelled through the US National Plant Germplasm System, on the one hand, and Seed Savers Exchange, on the other? How has the collection survived? And in what ways has it been lost?

**Figure 2:**
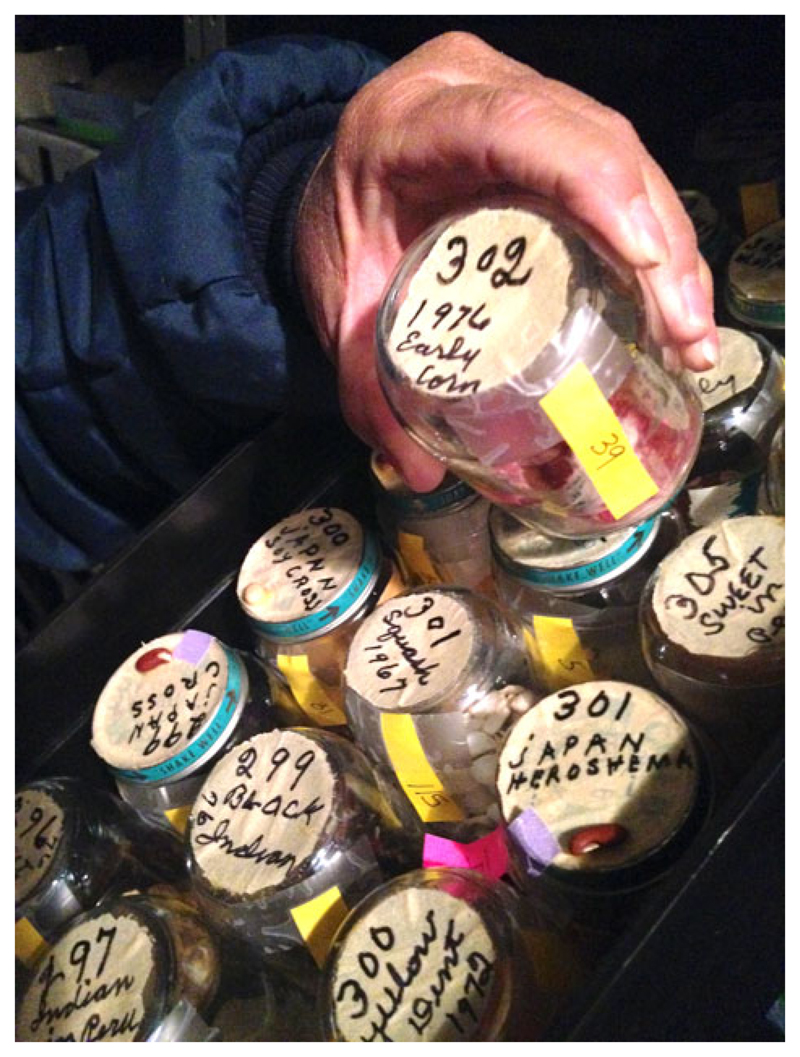
A tray containing Burt Berrier’s seed collection, still maintained in its original donated form at the National Laboratory for Genetic Resources Preservation, March 2017. Photo by author.

The NSSL, with its narrow vision of conservation through careful curation and cold storage, was not nearly as inept at dealing with the Berrier collection as Whealy had at times suggested. On a 2017 trip to the National Laboratory for Genetic Resources Preservation in Fort Collins, Colorado (the current incarnation of the National Seed Storage Laboratory) a former staff member was able to take me straight to the shelves bearing Berrier’s original baby-food jars, complete with masking-tape labels and glued-on beans, after a quick consultation of the database (see Figure 2). It took a little longer to locate the three file folders that documented, at least in part, the history that had brought the seeds to the bank and their subsequent lives in the National Plant Germplasm System, but a retired member of staff eventually achieved this too. Berrier’s donation had not been rejected, neglected or forgotten, but instead processed and incorporated into the national system – insofar as a system that typically seeks detailed information on provenance and potentially valuable agronomic characteristics could accommodate these atypical samples.

The process of accessioning a new sample into the national collection, especially one that comes with little information about its origin, can be years long. In the case of Berrier’s seeds, these were first entered into the Fort Collins database according to the esoteric numbering system deployed by Berrier (formalized in the database as a ‘Berrier number’). But to be assigned a ‘Plant Inventory’ or PI number, which means they are officially listed in the US Plant Inventory and maintained as part of the national collection, they had to be grown out, evaluated and increased such that there could be sufficient seeds available for distribution. The first task appears to have fallen to an employee of the then NSSL, Gene Keys, who attempted in the early 1980s to grow out as many types from the collection as could be salvaged. These were then shared with the relevant plant introduction stations for evaluation and perhaps further regeneration.^56^

Through the efforts of Keys and others, 110 of Berrier’s varieties have been assigned PI numbers since 1983. As of 8 September 2017, it was possible for a researcher anywhere in the world to log in to GRIN-Global, a database used by the US National Plant Germplasm System for cataloguing and distributing plant germplasm, and order a sample of twenty-five seeds for all but four of these bean accessions. He or she could also order samples of a hundred seeds each for six of eight maize varieties originating from Berrier’s material that have been regenerated and increased but not given PI numbers. Evidence of the origin of these accessions in Berrier’s collection lives on in the accession records. Many of these records have a transcription of Berrier’s handwritten labels in the database field ‘Plant name’, which is typically populated with a variety name or a breeders’ inventory number. You can search for and order beans he identified with a clear variety name, like ‘Dixie Butterpea Speckled Bush’ and ‘Bird Egg Pole Bean’, and also those he referred only by appearance or their source, such as ‘Pearl Ill [Illinois] Butter Bean’, ‘New Bean Gina’, or ‘Shiney’. Other samples whose common names or origins Berrier did not know are listed in the system by the number on the original jar label: ‘455’, ‘459 + 0’, or ‘2685 + 179’.^57^

As this indicates, Berrier’s bean collection continues in comparatively robust form in the freezers of the National Laboratory for Genetic Resources Preservation and the records of the US National Plant Germplasm System. Accessions like those derived from Berrier’s donation are not usually in high demand, so it is unlikely that they often make their way into fertile soil and still less likely that they are (or will ever be) tended by gardeners and enthusiasts such as Berrier and Withee. Although the fate that Whealy so vividly depicted in 1981 as a means of justifying the work of his organization and its transition to new modes was inaccurate, there is nonetheless a kernel of truth to the claim that Berrier’s collection was lost in its transfer to the NSSL. Access to the original collection itself is lost to all but researchers who know of its existence in the freezer. Access to regenerated materials with clear provenance in the collection (as through GRIN-Global) is wider but still limited in principle (though not always in practice) to professional researchers.

As I described, some of Berrier’s beans made their way from the NSSL into the Wanigan Associates collection not long after their deposition, and from there presumably into the central collection of Seed Savers Exchange. Their exact trajectories within these organizations and among their memberships are difficult to trace. Over the years, many members of Seed Savers Exchange offered varieties bearing the same common names as those that John Withee selected from Berrier’s collection. Current members continue to offer these today. But where did these come from? In all likelihood, they originated somewhere other than Berrier’s seeds, as many are old commercial varieties that once circulated widely. To date, I have come across only one bean collector – Russ Crow, also a member of Seed Savers Exchange from its earliest days – who claims to have a seed that came from Berrier’s collection through the work of John Withee. Because he lists it as ‘Berry’s Best’, a reference to Berrier and not one of the common names appearing in the extant records connected to Berrier’s original collection, it is difficult to link the seeds in Crow’s possession back to their specific progenitors.^58^ In other words, materials that were incorporated into Withee’s Wanigan Associates exchange network such as ‘Berry’s Best’ may well continue to be circulated, cultivated and enjoyed; however, without monitoring of their circulation or even persistent, standardized names, their connection to the original collection is severed. In this sense, too, Berrier’s collection is lost.

## Coda

There is a further set of sites where descendants of Berrier’s collection may yet survive. Whealy’s aim of creating reliable, long-term cold storage for Seed Savers Exchange eventually took shape in the construction of a ‘seed vault’ at the organization’s headquarters in Decorah, Iowa, as well as an arrangement for safety duplication at the current incarnation of the NSSL in Fort Collins. In addition, since 2007, Seed Savers Exchange has been further duplicating its collections for storage in the Svalbard Global Seed Vault, a safety duplication site buried beneath the permafrost on the Arctic island of Spitsbergen. Insofar as some of Berrier’s seeds were incorporated into the Seed Savers Exchange collection through their earlier incorporation into the Wanigan Associates collection, they may rest in these same storage facilities. This is where Whealy’s hope of using central storage as a way of securing collections from physical loss has ultimately led.^59^

The framework of cryopolitics formulated by Joanna Radin and Emma Kowal, with its emphasis on the perceived power over life conferred by low-temperature technologies, offers insight into why both state and grass-roots conservation efforts embraced (and continue to embrace) cold storage of seeds.^60^ Gathering, monitoring and slowing the decay of seeds through low temperatures appear to guarantee their future availability in a way that their messy, undocumented circulation as cultivated crops does not. And because the futures imagined by different seed conservators (such as staff at the US National Laboratory for Genetic Resources Preservation as compared to members of Seed Savers Exchange) may be radically different, one freezer is insufficient to secure them all.

At first glance, it seems unlikely that deep freeze in the Arctic permafrost was the fate that Berrier imagined for his seeds. Shortly before his death, he explained his collecting passion to Whealy, noting, ‘One thing about collecting beans, each has a life in it, it’s not dead as collecting clocks, dolls, guns, etc.’^61^ Seeds shipped to Svalbard are meant to be recalled only in the event of emergency. Extreme inaccessibility, a condition that runs counter to many of the explicit aspirations of Seed Savers Exchange, is in part what renders these last-resort materials safe, at least within the prevailing logic of salvage. If some descendants of Berrier’s collection are indeed resting below the permafrost, they are almost certainly there to die. Prolonging the life of seeds by sequestering them to cold storage, which in the end only delays their death, is precisely what is thought to ensure that seeds remain accessible, not to today’s cultivators but to tomorrow’s.

In looking to cold storage as a necessary fail-safe, both Berrier and Seed Savers Exchange (under Whealy’s direction) indicated that they had oriented their gaze away from the needs of their immediate community of growers and toward those of an imagined future population. Ensuring that collections were not lost through extinction for those growers-yet-to-come might well necessitate their occasional loss as accessible types to growers in the here and now. Among some seed savers, then, collections may be lost and yet persist – they may be dying in cold store for the ostensible purpose of ensuring life – a reminder that endings may be not only multiple (Bangham, Jardine and Kowal, this issue) but also contradictory.

## Footnotes

1 Quotation in Seed Savers Exchange (hereafter SSE) Winter Yearbook 1985, p. 1. All SSE publications were consulted with the assistance of the Robert Becker Memorial Library, Seed Savers Exchange, Decorah, Iowa.

2 True Seed Exchange (hereafter TSE) 1975, p. 1; 1975 invitation letter by Whealy, quoted in ‘How to set up a seed exchange’, *Mother Earth News*, July–August 1976, at www.motherearthnews.com/organic-gardening/seed-exchange-zmaz76jaztak, emphasis in original.

3 ‘F1 hybrid’ refers to ‘filial 1 hybrid’, the first-generation offspring resulting from the hybridization of two genetically distinct parent plants. F1 plants are often characterized by hybrid vigour, and for this reason sold by seed companies as superior types. However, the genetic mixing that generates hybrid vigour also means that the offspring of the F1 generation will be genetically heterogeneous – that is, they will not necessarily ‘grow true’ to perform as well as their F1 parent. This feature of F1 hybrids is a disadvantage for growers who cannot save the seed to use the next season, but a boon to seed companies who are guaranteed return customers.

4 TSE 1976, p. 17.

5 TSE 1976, p. 17.

6 TSE 1976, p. 1; Third Annual TSE 1978, p. 1.

7 On the role played by newsletters in facilitating exchange and generating community see Christopher M. Kelty, ‘This is not an article: model organism newsletters and the question of “open science”’, *BioSocieties* (2012) 7(2), pp. 140–168.

8 SSE 1981, p. 45.

9 SSE 1979, p. 1.

10 On the history of ‘genetic resources’ as a biological and agricultural concept see Christophe Bonneuil, ‘Seeing nature as a “universal store of genes”: how biological diversity became “genetic resources”, 1890–1940’, *Studies in History and Philosophy of Science Part C: Studies in History and Philosophy of Biological and Biomedical Sciences*, in press 2019, DOI: 10.1016/j.shpsc.2018.12.002. See also Marianna Fenzi and Christophe Bonneuil, ‘From “genetic resources” to “ecosystem services”: a century of science and global policies for crop diversity conservation’, *Culture, Agriculture, Food and Environment* (2016) 38(2), pp. 72–83.

11 Robin Pistorius, *Scientists, Plants, and Politics: A History of the Plant Genetic Resources Movement*, Rome: IPGRI, 1997; Robin Pistorius and Jeroen van Wijk, *The Exploitation of Plant Genetic Information: Political Strategies in Crop Development*, Wallingford: CABI, 1999; Tiago Saraiva, ‘Breeding Europe: crop diversity, gene banks, and commoners’, in N. Disco and E. Kranakis (eds.), *Cosmopolitan Commons: Sharing Resources and Risks across Borders*, Cambridge, MA: MIT Press, 2013, pp. 185–212; Sara Peres, ‘Saving the gene pool for the future: seed banks as archives’, *Studies in History and Philosophy of Science Part C: Studies in History and Philosophy of Biological and Biomedical Sciences* (2016) 55, pp. 96–104; Helen Anne Curry, ‘Breeding uniformity and banking diversity: the genescapes of industrial agriculture, 1935–1970’, *Global Environment* (2017) 10(1), pp. 83–113; Curry, ‘From working collections to the world germplasm project: agricultural modernization and genetic conservation at the Rockefeller Foundation’, *History and Philosophy of the Life Sciences* (2017) 39(5), DOI: 10.1007/s40656-017-0131-8.

12 See collected accounts of community-oriented seed saving initiatives in Carolyn Jabs, *The Heirloom Gardener*, San Francisco: Sierra Club, 1984; Virginia D. Nazarea, Robert E. Rhoades and Jenna E. Andrews-Swann (eds.), *Seeds of Resistance, Seeds of Hope: Place and Agency in the Conservation of Biodiversity*, Tucson: University of Arizona Press, 2013; Ronnie Vernooy, Pitambar Shrestha and Bhuwon Sthapit (eds.), *Community Seed Banks: Origins, Evolution and Prospects*, Abingdon: Routledge, 2015.

13 Scholarly accounts of Seed Savers Exchange and its approach to conservation include Michael K. Steinberg, ‘Valuing diversity: the role of “seed savers” in *in situ* crop plant conservation’, *Culture, Agriculture, Food and Environment* (2001) 23(3), pp. 41–45; Michael S. Carolan, ‘Saving seeds, saving culture: a case study of a heritage seed bank’, *Society & Natural Resources* (2007) 20(8), pp. 739–50; Nurcan Atalan Helicke, ‘Seed exchange networks and food system resilience in the United States’, *Journal of Environmental Studies and Sciences* (2015) 5(4), pp. 636–649. A recent account of the organization’s history by one of its founders is Diane Ott Whealy, *Gathering: Memoir of a Seed Saver*, Decorah, IA: Seed Savers Exchange, 2011.

14 For reflections on the histories and politics of cold-storage technologies, and their application to diverse purposes, see Joanna Radin and Emma Kowal (eds.), *Cryopolitics: Frozen Life in a Melting World*, Cambridge, MA: MIT Press, 2017.

15 For a sociological study that usefully brings together varied examples to offer a generalized picture of seed savers’ activities see Virginia D. Nazarea, *Heirloom Seeds and Their Keepers: Marginality and Memory in the Conservation of Biological Diversity*, Tucson: University of Arizona Press, 2005. See, similarly, Nazarea, Rhoades and Andrews-Swann, op. cit. (12). For recent accounts of seed savers in the United States see Brian Campbell, ‘Open-pollinated seed exchange: renewed Ozark tradition as agricultural biodiversity conservation’, *Journal of Sustainable Agriculture* (2012) 36(5), pp. 500–522; Helicke, op. cit. (13).

16 Laura Pottinger, ‘Planting the seeds of a quiet activism’, *Area* (2017) 49(2), pp. 215–222; Catherine Phillips, ‘Canada’s evolving seed regime: relations of industry, state, and seed savers’, *Environments* (2008) 36(1), pp. 5–18.

17 E.g. Paul Robert Gilbert, ‘Deskilling, agrodiversity, and the seed trade: a view from contemporary British allotments’, *Agriculture and Human Values* (2013) 30(1), pp. 101–114; Helicke, op. cit. (13); Phillips, op. cit. (16).

18 This is in contrast to the burgeoning literature on the history of state-led genetic conservation programs; see references in notes 10 and 11.

19 E.g. Michael S. Carolan, ‘Conserving nature, but to what end? Conservation policies and the unanticipated ecologies they support’, *Organization & Environment* (2006) 19(2), pp. 153–170; Thom van Dooren, ‘Banking seed: use and value in the conservation of agricultural diversity’, *Science as Culture* (2009) 18(4), pp. 373–395. Both of these authors rightly point out the more encompassing notions of what is to be conserved that dominate in seed-saving organizations and the different conservation practices that emerge; I highlight instead how different conservation aims could lead to similar conservation practices.

20 On the back-to-the-land and homesteading movements of this period see Ryan H. Edgington, ‘“Be receptive to the Good Earth”: health, nature, and labor in countercultural back-to-the-land settlements’, *Agricultural History* (2008) 82(3), pp. 279–308; Dona Brown, *Back to the Land: The Enduring Dream of Self-Sufficiency in Modern America*, Madison: University of Wisconsin Press, 2011, Chapter 7.

21 A recent institutional account is found in Whealy, op. cit. (13).

22 ‘The plowboy interview: Kent Whealy’, *Mother Earth News*, January–February 1982, at www.motherearthnews.com/nature-and-environment/interview-with-seed-savers-exchange-founder. Harlan, a botanist, breeder and USDA plant explorer, and Garrison Wilkes, a botanist with particular expertise in maize diversity, were two particularly vocal American scientists on the subject of genetic erosion in the early 1970s.

23 ‘The plowboy interview: Kent Whealy’, op. cit. (22). The article by Ehrlich to which Whealy referred appears to have been ‘Paul Ehrlich interview: the population bomb’, *Mother Earth News*, July–August 1974, at www.motherearthnews.com/nature-and-environment/paul-ehrlich-the-population-bomb-zmaz74jazraw.

24 SSE 1981, pp. 44–46.

25 TSE 1976, pp. 6, 9.

26 Third Annual TSE 1978, pp. 4, 5.

27 SSE 1979, p. 7.

28 USDA, *The National Plant Germplasm System*, Program Aid no 1188, Washington, DC: USDA, 1977, p. 4. See also Sam Burgess (ed.), *The National Program for Conservation of Crop Germplasm (A Progress Report on Federal/State Cooperation)*, Athens, GA: University Printing Department, 1971.

29 Letter from L. Bass, quoted in Third Annual TSE 1978, p. 28.

30 E.g. SSE Winter 1986, pp. 39, 42; SSE Winter 1987, pp. 53, 57.

31 See correspondence in the records of the National Laboratory for Genetic Resources Preservation (hereafter NLGRP), Fort Collins, Colorado, folder ‘Withee, J. (Beans)’. For a further account of John Withee see ‘The life and legacy of the Bean Man’, at www.seedsavers.org/withee-exhibit-bean-man.

32 SSE 1979, p. 22.

33 Dietz to Bass, 4 January 1976, NLGRP, folder ‘Berrier Bean Collection 2’.

34 The descriptors ‘bush’ and ‘pole’ refer to the morphology of the plant: ‘bush’ beans typically grow a meter or less in height and support their own weight while ‘pole’ beans grow as vines and require a stake for support. Berrier to Bass, 8 January 1977, NLGRP, folder ‘Berrier Bean Collection 2’.

35 Beans were and are a popular focus for seed savers who amass large collections of a single vegetable. One reason for this is that a great deal of diversity is visible in the seeds themselves, which can differ dramatically in shape, size and colour. Many other vegetables must be grown out in order for their diversity to be similarly appreciated.

36 Bass to M. Berrier, 17 February 1978, NLGRP, folder ‘Berrier Bean Collection 2’.

37 SSE 1979, p. 22.

38 E.g. Bass to Lillibridge, 30 March 1978; Bass to Stokes, 11 May 1978, NLGRP, folder ‘Berrier Bean Collection 2’.

39 Bass to Stokes, 11 May 1978, NLGRP, folder ‘Berrier Bean Collection 2’.

40 Bass to Withee, 29 March 1978, NLGRP, folder ‘Withee, J. (Beans)’.

41 ‘Beans from the Berrier Collection (for regrowing)’, 5 February 1979, folder ‘Berrier Bean Collection 3’.

42 ‘Beans from the Burt F. Berrier Collection [with annotation by Withee]’, [1978], NLGRP, folder ‘Withee, J. (Beans)’.

43 SSE 1979, p. 32.

44 ‘The life and legacy of the Bean Man’, op. cit. (31), emphasis added.

45 SSE 1981, pp. 48, 51–52.

46 SSE 1981, p. 52.

47 SSE Harvest Edition 1994, p. 129.

48 SSE Harvest Edition 1994, p. 130–131.

49 SSE 1981, p. 46.

50 SSE 1981, p. 47.

51 SSE Harvest Edition 1987, pp. 93–96.

52 SSE Harvest Edition 1987, p. 96.

53 SSE Harvest Edition 1987, p. 96.

54 On plant exploration within the US Department of Agriculture see Karen A. Williams, ‘An overview of the U.S. National Plant Germplasm System’s exploration program’, *HortScience* (2005) 40(2), pp. 297–301.

55 SSE Harvest Edition 1987, p. 96.

56 The history is not entirely clear; this is my best reconstruction from the available documents. See NLGRP, ‘Berrier Bean Collection’ files.

57 A search portal for the USDA collections cataloged in GRIN-Global can be accessed at npgsweb.ars-grin.gov/gringlobal/search.aspx.

58 Russ Crow, ‘A bean collector’s window’, 2012–2017, www.abeancollectorswindow.com/index.html. The exchange listings of Seed Savers Exchange can be searched at exchange.seedsavers.org.

59 Not without objections from Whealy, who left Seed Savers Exchange in 2007. See Kent Whealy, ‘Response regarding Svalbard’, November 2010, at www.centerforfoodsafety.org/files/response-final_40147.pdf.

60 Radin and Kowal, op. cit. (14); for a contribution that deals explicitly with seed banks see Thom van Dooren, Chapter 13, ‘Banking the forest: loss, hope, and care in Hawaiian conservation’, in Radin and Kowal, op. cit. (14).

61 Third Annual TSE 1978, p. 18.

